# Rheumatoid arthritis and cancer risk in the Million Women Study

**DOI:** 10.1093/ije/dyae006

**Published:** 2024-02-29

**Authors:** TienYu Owen Yang, Sarah Floud, Gillian K Reeves, Million Women Study Collaborators, Simon Abbott, Rupert Alison, Sarah Atkinson, Krys Baker, Angela Balkwill, Isobel Barnes, Judith Black, Roger Blanks, Anna Brown, Andrew Chadwick, Dave Ewart, Sarah Floud, Kezia Gaitskell, Toral Gathani, Laura Gerrard, Adrian Goodill, Carol Hermon, Sau Wan Kan, Nicky Langston, Keren Papier, Kirstin Pirie, Gillian Reeves, Keith Shaw, Emma Sherman, Karl Smith-Byrne, Helena Strange, Sian Sweetland, Ruth Travis, Lyndsey Trickett, Clare Wotton, Owen Yang Heather Young, Emily Banks, Lucy Carpenter, Carol Dezateux, Sarah Floud, Julietta Patnick, Richard Peto, Gillian Reeves, Cathie Sudlow

**Affiliations:** Cancer Epidemiology Unit, Nuffield Department of Population Health, University of Oxford, Oxford, UK; Cancer Epidemiology Unit, Nuffield Department of Population Health, University of Oxford, Oxford, UK; Cancer Epidemiology Unit, Nuffield Department of Population Health, University of Oxford, Oxford, UK

**Keywords:** Epidemiology, rheumatoid arthritis, smoking, cancer

## Abstract

**Background:**

Most previous studies of rheumatoid arthritis (RA) and cancer risk have lacked information on potential confounding factors. We investigated RA-associated cancer risks in a large cohort of women in the UK, taking account of shared risk factors.

**Methods:**

In 1996–2001, women aged 50–64, who were invited for routine breast screening at 66 National Health Service (NHS) screening centres in England and Scotland, were also invited to take part in the Million Women Study. Participants provided information on sociodemographic, lifestyle and health-related factors, including RA, and were followed up for cancers and deaths. Cox regression yielded RA-associated hazard ratios (HRs) of 20 cancers, adjusted for 10 characteristics including smoking status and adiposity.

**Results:**

Around 1.3 million women (half of those invited) were recruited into the study. In minimally adjusted analyses, RA was associated with the risk of 13 of the 20 cancers. After additional adjustment for lifestyle factors, many of these associations were attenuated but there remained robust evidence of RA-associated increases in the risk of lung (HR 1.21, 95% confidence interval 1.15–1.26), lymphoid (1.25, 1.18–1.33), myeloid (1.12, 1.01–1.25), cervical (1.39, 1.11–1.75) and oropharyngeal (1.40, 1.21–1.61) cancers, and decreases in the risk of endometrial (0.84, 0.77–0.91) and colorectal (0.82, 0.77–0.87) cancers.

**Conclusions:**

After taking account of shared risk factors, RA is positively associated with lung and certain blood and infection-related cancers, and inversely associated with colorectal cancer. These findings are consistent with existing hypotheses around immune response, susceptibility to infections, and chronic inflammation. The inverse association observed for endometrial cancer merits further investigation.

Key Messages

- This is the first large prospective study to examine associations of rheumatoid arthritis (RA) with a range of site-specific cancers, taking account of shared risk factors and thereby helping to elucidate which cancers are most likely to be causally related to RA (or its treatment).
- After adjustment for shared risk factors, there remained robust evidence of RA-associated increases in the risk of lung, lymphoid, myeloid, cervical and oropharyngeal cancers, and decreases in the risk of endometrial and colorectal cancers.
- Most of the observed associations between RA and specific cancers are consistent with existing hypotheses related to immune response, susceptibility to infection, and chronic inflammation; the apparent inverse association with endometrial cancer is novel and merits further investigation.

## Introduction

There is increasing evidence that individuals with rheumatoid arthritis (RA) are at greater or lesser risk of developing certain cancers compared with the general population. The most consistently reported associations have been with an increased risk of lung and blood cancer,^1–10^ but some studies and meta-analyses have also reported differences in the risk of female cancers, cancers of the digestive system and skin cancer.^3^^,^^5^^,^^10–21^

RA and its treatment lead to immune dysregulation and susceptibility to infections that might plausibly affect the risk of multiple cancers, and a clearer understanding of the relationship between RA and cancer is of clinical and pharmaceutical interest. However, RA is known to be associated with some of the main risk factors for cancer, including smoking and obesity,^22^^,^^23^ and so some of the associations observed between RA and specific cancers could be due to confounding. Since much of the existing evidence on RA and cancer is based on routine linkage studies with limited information on sociodemographic and lifestyle factors, further large prospective studies with information on all relevant confounders are needed to assess whether associations between RA and specific cancers are likely to be causal.

We investigated associations of RA with the risk of 20 specific cancers in a cohort of 1.3 million middle-aged women in the UK, taking careful account of potential confounding by sociodemographic, anthropometric and lifestyle factors.

## Methods

### Study population and definitions of exposure and outcome

Between 1996 and 2001, the Million Women Study recruited 1.3 million UK women aged 50–64 when they were invited to routine screening through the National Health Service (NHS) Breast Screening Programme. Participants provided information on sociodemographic, anthropometric, lifestyle and health-related factors and were followed up through electronic linkage to routinely collected NHS data on deaths, hospital admissions and cancer registrations; further details are given in the cohort profile^24^ and at [https://www.ceu.ox.ac.uk/research/the-million-women-study]. All participants gave written consent for participation.

The study included about a quarter of UK women in the eligible age range (50–64 years) at the time of recruitment. Participants were slightly less likely to be in the most deprived national quintile than women of a similar age in the general population, but were broadly similar in many other respects.^24^

Classification of participants according to their status with respect to RA at recruitment in median year 1998 (range 1996–2000) was based on their response to the question: ‘Are you now being treated for rheumatoid arthritis (yes/no)?’.

Classification of cancers by site was based on the International Classification of Diseases, 10th Revision (ICD-10). The International Classification of Diseases for Oncology, Morphology of Neoplasms, 3rd Edition, ICD-O-3, was used to categorize blood cancers according to the World Health Organization classification 2008^25^ and to subdivide oesophageal cancers by histological subtype.

### Statistical analysis

Hazard ratios of 20 site-specific cancers were estimated in women who reported having been treated for RA at recruitment and compared with those who did not. Women were excluded if they had any cancer registration prior to recruitment (other than non-melanoma skin cancer, ICD-10 C44); otherwise they contributed person-years from their date of recruitment to date of cancer registration (other than non-melanoma skin cancer), death or end of follow-up (31 December 2018).

For endometrial cancer and ovarian cancer, women who reported having had a hysterectomy and/or bilateral oophorectomy at recruitment, respectively, were excluded from analyses.

Cox regression models were used to estimate hazard ratios (HRs) for each cancer. The underlying time variable was attained age, and analyses were stratified by year of recruitment, year of birth (in 3-year intervals) and 10 regions of residence, with adjustment for 10 baseline characteristics: residential area-derived social deprivation (based on Townsend Index,^26^ in fifths); education (did and did not complete compulsory education, and any technical, secondary or tertiary qualifications); joint status with respect to smoking (never, past, current: <5, 5–9, 10–14,15–19, 20–24, 25+ cigarettes per day) and alcohol consumption (0, 1, 2–4, 5–9, 10–19, 20+ units per week); body mass index (BMI) (<22.5, 22.5–24.9, 25.0–27.4, 27.5–29.9, 30.0–32.4, 32.5–34.9, 35.0–37.4, 37.5+ kg/m^2^); strenuous exercise (rarely, <1, 1, 2–3, 4–6, 7 days a week); age at menopause (premenopausal, postmenopausal with age at menopause: <50, 50–54, 55+ years) and use of menopausal hormone therapy (never, past, current); duration of past oral contraceptive use (0, 1–5, 6–11 and 12+ years); age at first birth (<24 and 24+ years) and parity (0, 1, 2, 3+); and age at menarche (<12, 12–14, 15+ years). Women with missing values for any adjustment variable were assigned to a separate category for that variable (<6% for each variable).

To assess the extent to which associations of cancer risk with RA were likely to be due to confounding with 10 sociodemographic, anthropometric and lifestyle factors, we examined the change in the RA-attributable chi-square statistic after adjustment for each of these factors in turn, and after adjustment for all 10 factors.^27^ Where the RA-associated chi-square statistic reduced by two-thirds or more on adjustment for all 10 factors, the adjusted association was deemed to be susceptible to substantial residual confounding. We also conducted analyses restricted to women who had never smoked.

Additional sensitivity analyses were conducted to assess the likely impact of other potential sources of bias. To assess the impact of possible misclassification of arthritis types using self-reported RA, a sensitivity analysis was conducted which excluded women who also reported having osteoarthritis in the same section of the questionnaire at study baseline. Since early symptoms of cancer might affect behavioural risk factors such as BMI, and the likelihood of having other conditions diagnosed, we repeated analyses after excluding the first 5 years of follow-up. We also assessed the relationship of RA with the risk of breast, endometrial, ovarian and cervical cancer in never-users of menopausal hormone therapy. To assess the impact of missing information on potential confounders, we repeated the main analysis restricted to women with known values of all adjustment factors. Last, we assessed the potential impact of more general misclassification in self-reported RA status by conducting analyses using a more specific but less inclusive criterion for RA ascertainment in which women were classed as having RA only if they self-reported RA and had a diagnosis of RA (ICD-10 M05 and M06) recorded in at least one hospital admission at any time.

## Results

After excluding 50430 women with a prior cancer registration, there were 1313838 women eligible for inclusion in the analysis. Characteristics of the included women, according to RA status, are shown in Table 1. Women who reported being treated for RA at baseline (*n* = 62681) were much more likely to be smokers, to be obese, to engage less in strenuous exercise and to consume less alcohol.

**Table 1.** Characteristics of participants at study baseline

	Women who self-reported being treated for RA at recruitment	All others
***N***	62 681	1 251 157
**Mean age at recruitment in years (SD)**	57.9 (4.9)	56.6 (4.8)
		
**Most deprived fifth, *n* (%)**	20 632 (33%)	242 651 (19%)
**Have any educational qualification, *n* (%)**	23 620 (39%)	694 160 (57%)
		
**Current smoker, *n* (%)**	16 105 (26%)	238 058 (19%)
**Alcohol intake >5 units per week, *n* (%)**	13 451 (22%)	392 776 (32%)
**Strenuous exercise at least once a week, *n* (%)**	15 529 (25%)	475 748 (39%)
**Body mass index >30 kg/m^2^, *n* (%)**	16 488 (28%)	205 791 (17%)
		
**Menarche later than 14 years old, *n* (%)**	12 444 (20%)	206 237 (17%)
**Ever used oral contraceptives >1 year, *n* (%)**	27 716 (44%)	646 982 (52%)
**Number of children, mean (SD)**	2.4 (1.5)	2.2 (1.2)
**Ever used menopausal hormone therapy, *n* (%)**	32 875 (53%)	620 505 (50%)
		
**Mean follow up in years (SD)**	16.8 (5.5)	17.7 (4.9)
**Cancer cases, *n* (%)**	12 959 (21%)	238 217 (19%)
**Mean time to cancer diagnosis from baseline in years (SD)**	10.8 (5.7)	10.8 (5.7)
		

RA: rheumatoid arthritis; SD: standard deviation

**Figure 1.**
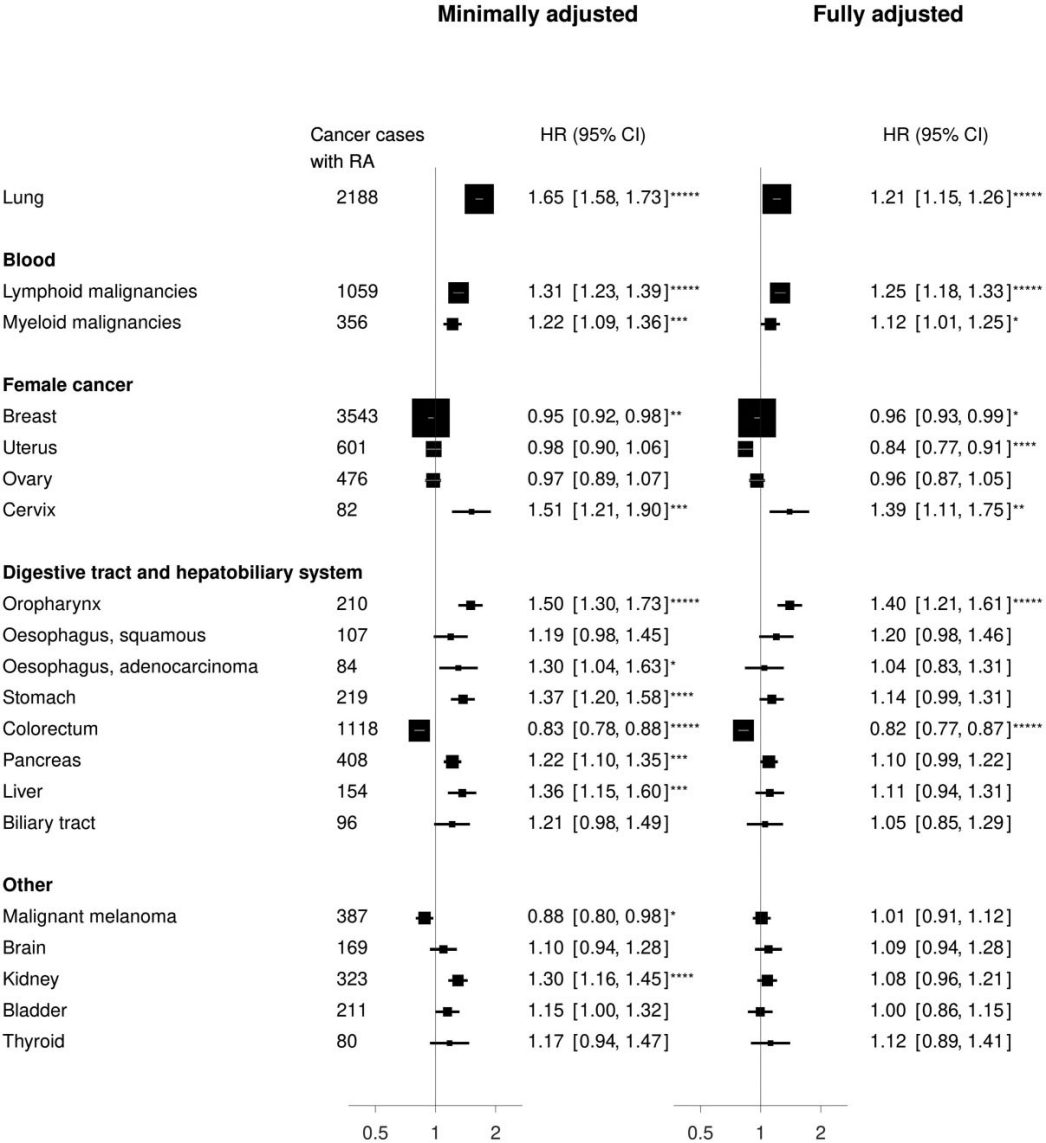
Hazard ratio of cancer in individuals with rheumatoid arthritis (RA) compared with others in the Million Women Study. Numbers and forest plots show hazard ratios (HR) and 95% confidence intervals. Minimal adjustment includes year of recruitment; year of birth; 10 regions of residence; age. Full adjustment includes year of recruitment; year of birth; 10 regions of residence; age; social deprivation; education; joint alcohol consumption and smoking status; body mass index; strenuous exercise; age at menarche; age at first birth and parity; duration of oral contraceptive use; menopausal status and age; use of menopausal hormone therapy. Number of asterisks (*) indicates level of statistical significance (*<0.05; **<0.005; ***<0.0005; ****<0.00005; *****<0.000005)

After controlling for age, year of birth, year of recruitment and region, there was evidence of an association of RA (at the *P *<0.05 level) with 13 of the 20 cancers considered (Figure 1). However, after adjustment for additional confounders, many of these associations were attenuated. Evidence of an RA-associated increase in risk remained, based on fully adjusted hazard ratios (HRs), for lung cancer (1.21, 95% confidence interval 1.15–1.26), lymphoid malignancies (1.25, 1.18–1.33), myeloid malignancies (1.12, 1.01–1.25), cervical cancer (1.39, 1.11–1.75), and oropharyngeal cancer (1.40, 1.21–1.61). There was also an associated decrease in the risk of endometrial (0.84, 0.77–0.91) and colorectal cancer (0.82, 0.77–0.87), and a modest decrease in the risk of breast cancer (0.96, 0.93–0.99).

For many cancers, adjustment for lifestyle factors considerably reduced the RA-associated chi-square statistic, with the biggest reductions due to adjustment for social deprivation, smoking and adiposity (Supplementary Table S1, available as Supplementary data at *IJE* online). Of the eight cancers for which there remained evidence of an association after full adjustment, the only one for which the chi-square statistic was reduced by two-thirds or more after adjustment was lung cancer, with an 86% reduction in the chi square statistic, and hence this association was deemed to be susceptive to residual confounding.

In analyses restricted to never-smokers, there was a similar association of RA with lung cancer risk (1.31, 1.10–1.56) (Supplementary Figure S1, available as Supplementary data at *IJE* online). The only other notable changes after restriction to never-smokers were an increase in the RA-associated HRs for kidney cancer [from 1.08 (0.96–1.21) to 1.23 (1.03–1.47)] and for breast cancer [from 0.96 (0.93–0.99) to 1.00 (0.95–1.06)], although these are difficult to interpret and could be due to chance.

Unexpectedly, among the seven cancer sites for which there was little evidence of an association with RA after minimal adjustment, a reduction in endometrial cancer in women with RA was observed after adjustment for lifestyle factors [HR changed from 0.98 (0.90–1.06) to 0.84 (0.77–0.91)], and this was predominantly due to adjustment for BMI (HR = 0.80 after adjustment for BMI only, Supplementary Table S1, available as Supplementary data at *IJE* online).

**Figure 2.**
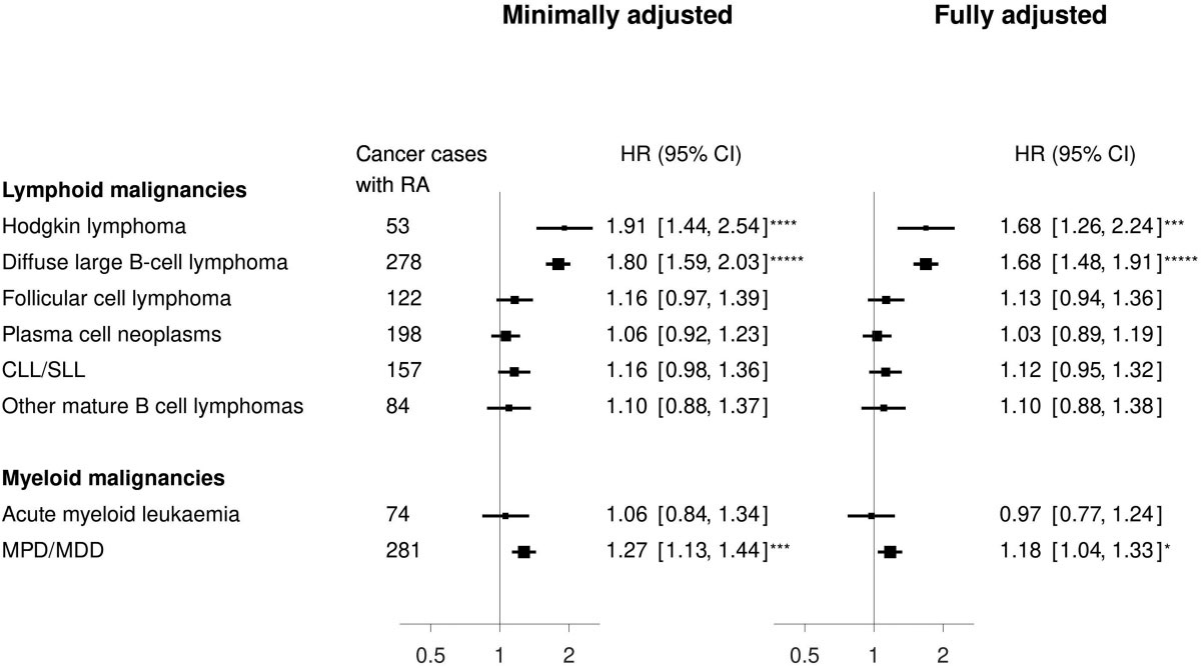
Hazard ratio of haematological malignancies by histological subtypes in individuals with rheumatoid arthritis (RA) compared with others in the Million Women Study. Numbers and forest plots show hazard ratios (HR) and 95% confidence intervals. Minimal adjustment includes year of recruitment; year of birth; 10 regions of residence; age. Full adjustment includes year of recruitment; year of birth; 10 regions of residence; age; social deprivation; education; joint alcohol consumption and smoking status; body mass index; strenuous exercise; age at menarche; age at first birth and parity; duration of oral contraceptive use; menopausal status and age; use of menopausal hormone therapy. Number of asterisks (*) indicates level of statistical significance (*<0.05; **<0.005; ***<0.0005; ****<0.00005; *****<0.000005). CLL/SLL, chronic lymphocytic leukaemia (CLL) and small lymphocytic lymphoma; MPD/MDD, myeloproliferative or myelodysplastic disorders

The overall association of RA with all lymphoid cancers after adjustment (HR = 1.25, 1.18–1.33) appeared to be largely driven by comparatively greater associations with Hodgkin lymphoma (1.68, 1.16–2.24) and diffuse large B-cell lymphoma (1.68, 1.48–1.91), as there was little evidence of any material association with other common subtypes (Figure 2). For myeloid cancers, there was a small increase in the risk of malignant myeloproliferative or myelodysplastic disorders (1.18, 1.04–1.33) but no evidence of an association with acute myeloid leukaemia (0.97, 0.77–1.24).

The findings in Figure 1 were not materially affected after excluding 12 118 (19%) women who also reported osteoarthritis in the same section of questionnaire at study baseline (Supplementary Figure S2, available as Supplementary data at *IJE* online); after excluding the first 5 years of follow-up (Supplementary Figure S3, available as Supplementary data at *IJE* online); or after restriction of analyses of female cancers to women who reported never having used menopausal hormone therapy at recruitment (Supplementary Figure S4, available as Supplementary data at *IJE* online). The findings were also similar after restriction to women with complete information on all confounders, in that no hazard ratio was altered by more than 9% (Supplementary Figure S5, available as Supplementary data at *IJE* online).

In analyses in which women were classed as having RA only if they both self-reported RA and had RA recorded in at least one hospital admission, the overall pattern of associations was similar although some associations between RA and site-specific cancers became more marked. The largest change in the magnitude of any association was for lymphoid malignancies [from 1.25 (1.18–1.33) to 1.79 (1.63–1.97)] (Supplementary Figure S6, available as Supplementary data at *IJE* online).

## Discussion

To our knowledge, this study, which included some 62 000 women with RA, over 12 000 of whom were diagnosed with cancer, is the largest investigation of RA and cancer to take account of potential confounding by lifestyle factors. The findings demonstrate that, even after taking account of smoking, BMI and other factors, women with RA have a higher risk of lung cancer, Hodgkin lymphoma, diffuse large B-cell lymphoma, myeloid malignancies, cervical cancer and oropharyngeal cancer, and a lower risk of endometrial and colorectal cancer. The greatest associations of RA with risk were for Hodgkin and diffuse large B-cell lymphoma, for which women with RA appeared to be at a 60% increased risk. The findings for each cancer are discussed below in the context of the existing evidence.

### Lung cancer

Although RA is known to cause interstitial lung inflammation, mechanisms for a smoking-independent pathway which could explain an association of RA with lung cancer are under-investigated.^8^^,^^9^^,^^11^ A recent meta-analysis of 11 population-level cohort studies, including 1365 lung cancer cases, reported a pooled relative risk of lung cancer of 1.44, 95% CI 1.31–1.57,^8^ but the majority of contributing studies did not adjust for smoking. In the same report, a Mendelian randomization study, which used data from the International Lung Cancer Consortium, found that a genetic predisposition score for RA was not associated with an increased risk of lung cancer.^8^ In our analyses, which included more lung cancer cases than any previous observational study, we observed a modest increase in the risk of lung cancer after careful adjustment for smoking. We also found a similar association in women who reported never smoking (HR 1.31, 1.10–1.56) which has not been previously demonstrated and suggests that there is a small smoking-independent effect of RA on lung cancer.

### Blood cancers

The increased risk of lymphoid and myeloid malignancies in women with RA observed here is consistent with previous meta-analyses of earlier and smaller studies. In such meta-analyses, the pooled standardized incidence ratio (SIR) for lymphoma was 2.46 (95% CI 2.05–2.96), based on 12 studies including 1102 lymphomas,^11^ and the incidence rate ratio for leukaemia was 1.51 (95% CI 1.34–1.70), based on 14 studies.^7^ Given the considerably larger number of cases and histology information available in our analysis, we were able to provide further evidence regarding the specificity of those associations to particular subtypes based on pre-defined blood cancer classifications. These analyses revealed that RA was primarily associated with Hodgkin lymphoma, diffuse large B-cell lymphoma and malignant myeloproliferative/myelodysplastic disorders. The RA-associated risk of blood cancer is thought to be related to the dysregulation of the immune system, perhaps more from the disease than from the treatment.^4^^,^^28^^,^^29^ To our knowledge there is no known single pathway or treatment linked to the three subtypes. Whether this reflects single or multiple immune pathways, or reflects RA or its treatment, is beyond the scope of this report.

### Female cancers

This is by far the largest investigation of RA in relation to cancers of the breast, endometrium, ovary and cervix. A recent meta-analysis of RA and breast cancer found a non-significant decrease in risk (SIR = 0.86, 95% CI 0.73–1.01) based on ∼3000 RA patients with breast cancer, but this association was not adjusted for potential confounders.^11^^,^^30^ An analysis of UK Biobank data, including 5939 RA patients, reported a lower risk (HR = 0.64, 95% CI 0.46–0.89) which was adjusted for additional lifestyle factors but not hormonal factors.^31^ The RA-associated reduction in breast cancer risk observed here, based on 3543 RA patients with breast cancer, was comparatively modest (HR = 0.96, 0.93–0.99) and was not evident in never-smokers. The difference in the magnitude of previously reported associations of RA with breast cancer with those observed here may partly reflect greater control for confounding.

In contrast, we found a lower risk of endometrial cancer in women with RA (HR = 0.84, 0.77–0.91) which only became apparent after adjustment for BMI. A reduction in the crude hazard ratio of endometrial cancer has been reported in record linkage studies based on RA patients in the Swedish inpatient RA register, identified in 1990–2003 (84 cancers, SIR = 0.60, 95% CI 0.48–0.75),^32^ and in 1980–2004 (117 cancers, SIR = 0.66, 95% CI 0.55–0.7)^21^; and in a similar study of California inpatient RA records (126 cancers, SIR = 0.53, 95% CI 0.44–0.63).^20^ A record linkage study in Taiwan also reported a consistent, though non-significant, decrease in risk (32 cases, SIR = 0.84, 95% CI 0.60–1.19).^16^ It is unclear as to whether this apparent reduction in endometrial cancer risk among women with RA is likely to be related to the treatment or to the disease itself.

A previous meta-analysis found no association of RA with cervical cancer (297 cancers in 15 studies, SIR = 0.87, 95% CI 0.72–1.05).^11^ Our finding of an increased risk (HR = 1.39, 1.11–1.75) therefore requires confirmation in subsequent studies. Cervical cancer is infection-related and largely preventable, and our finding may be related to a general observation that RA is associated with a higher rate of general infection^12^ and perhaps specifically of human papillomavirus virus (HPV) infection.^33^ Since we did not have information on HPV status, and women in this study were older than the average age at cervical cancer diagnosis, it is difficult to know how to interpret this finding. Further studies will be required to confirm if this association is causal, and whether it is likely to be explained by differences in infection patterns among individuals with RA.

### Cancers of the digestive system

Coincidental to the finding for cervical cancer, we also found a novel RA-associated increase in risk of oropharyngeal cancer which was effectively unchanged when analyses were restricted to never-smokers. There is increasing evidence that HPV infection has a major role in the development of oropharyngeal cancer,^34^ and this excess risk could potentially be explained by higher rates of infection with HPV, and possibly other pathogens, in women with RA.

There was little evidence of an association of RA with other cancers of the digestive and biliary tract (oesophagus, stomach, liver, biliary tract and pancreas) after taking account of potential confounders, particularly smoking and obesity. One exception to this was the observed reduction in risk of colorectal cancer in women with RA (0.82, 0.77–0.87). This association has been reported before,^11^ and has been attributed to the effect of anti-inflammatory treatment for RA, akin to the protective association which has been observed in long-term aspirin users.

### Strengths and limitations

The main strengths of this study are the extremely large sample size, long follow-up and prospectively collected information on common lifestyle factors. This allowed for a systematic investigation of RA in relation to subsequent risk of many cancers, so that biases due to preferential publication of selected findings and/or retrospective recall were minimized. Equally important was the ability to control for the association of RA with major risk factors for cancer, especially smoking and adiposity. Nevertheless this is an observational study, and some degree of residual confounding due to unmeasured factors, to adjustment for imperfect measures of known risk factors or to informative censoring related to these factors,^35^ cannot be ruled out. For this reason, we used various criteria to assess the extent to which associations were likely to have been influenced by residual confounding, and highlighted only those that appeared relatively robust to adjustment and to various sensitivity analyses.

Our analyses were based on self-reported RA, which has the advantage of affording all women the opportunity to have prevalent RA recorded but may be subject to some degree of misclassification. However, in sensitivity analyses in which women were classed as having RA only if they both self-reported RA and had at least one mention of RA in their hospital admission records, the overall pattern of RA-associated risks appeared similar, and the results were not affected after exclusion of women who reported having both RA and osteoarthritis at study baseline. We were unable to investigate the extent to which the observed associations, or lack of associations, might have been influenced by disease subtype, duration and severity or by treatment, because such information was not available. Despite being the largest study to date to take account of potential confounding by lifestyle factors, case numbers for some rare cancers were relatively small and so findings may still be susceptible to chance or residual confounding. Since our study included only women, we were also unable to study the association of RA with cancer in men.

### Conclusions

Our findings suggest that, even after allowing for shared risk factors, RA is associated with a higher risk of lung cancer, certain blood cancers, cervical cancer and oropharyngeal cancer, and with a lower risk of endometrial and colorectal cancer. Associations of RA with blood cancers and colorectal cancer have been reported previously, and are in line with existing hypotheses regarding immune dysregulation and chronic inflammation, but the observed associations with cervical, oropharyngeal and endometrial cancer are novel and warrant further investigation.

## Ethics approval

Ethical approval for the study was provided by the Oxford and Anglia Multi-Centre Research Ethics Committee (97/5/001).

## Supplementary Material

dyae006_Supplementary_Data

## Data Availability

The Million Women Study data sharing policy can be accessed online: [https://www.ceu.ox.ac.uk/research/the-million-women-study/data-access-and-sharing/data-access-policy].
