# Supplementary material for: Rheumatoid arthritis and cancer risk in the Million Women Study
Source: Int J Epidemiol. 2024 Feb 29;53(2):dyae006. doi: 10.1093/ije/dyae006 (PMC10904146; doi:10.1093/ije/dyae006)

Rheumatoid arthritis and cancer risk in the Million Women Study

TienYu Owen Yang[1]; Sarah Floud[1]; Gillian K Reeves[1] for the Million Women Study collaborators
[1]Cancer Epidemiology Unit, Nuffield Department of Population Health, University of Oxford, UK

Correspondence to Dr. TienYu Owen Yang
Richard Doll Building, Old Road Campus, Oxford OX3 7LF UK
Owen.yang@ndph.ox.ac.uk

Supplementary Materials

Table of Contents

TABLE S1. EFFECT OF ADJUSTMENT 2
FIGURE S1. NEVER-SMOKERS 3
FIGURE S2. EXCLUDING INDIVIDUALS WHO REPORTED ALSO HAVING OSTEOARTHRITIS 4
FIGURE S3. EXCLUDING FIRST FIVE YEARS OF FOLLOW UP 5
FIGURE S4. EXCLUDING PARTICIPANTS WHO HAD EVER USED MENOPAUSAL HORMONE THERAPY 6
FIGURE S5. EXCLUDING PARTICIPANTS WITH MISSING INFORMATION 7
FIGURE S6. USE OF ALTERNATIVE DEFINITION OF SELF-REPORTED RHEUMATOID ARTHRITIS WHICH ADDITIONALLY REQUIRES A DIAGNOSIS OF RHEUMATOID ARTHRITIS IN HOSPITAL RECORDS 8

Table S1. Effect of adjustment

Hazard ratio of cancer associated with rheumatoid arthritis: effect of adjustment, separately and jointly, for potential confounding factors

Percentages underneath the hazard ratio show reduction in chi-square value attributable to rheumatoid arthritis compared to minimal adjustment. Asterisks (*) mark hazard ratios which are significant at *P*<0.05 level.

	**Hazard ratio (change in RA associated chi-square statistic after each additional adjustment)**												
	**Adjusted for age, year of birth, year of recruitment, and region only**	**Additionally and separately adjusted for** Education	Social deprivation	Age at menarche	Number of children and age at first birth	Use of oral contraceptives	Menopausal status and use of menopausal hormone therapy	Smoking	Alcohol consumption	Smoking and alcohol consumption	Body mass index	Exercise	**Adjusted for all**
Lung	**1.65***	1.49*<br>(-37%)	1.46*<br>(-42%)	1.64*<br>(-4%)	1.54*<br>(-25%)	1.65*<br>(0%)	1.59*<br>(-15%)	1.30*<br>(-71%)	1.62*<br>(-8%)	1.28*<br>(-74%)	1.70*<br>(+9%)	1.53*<br>(-27%)	**1.21*<br>(-85%)**
Lymphoid malignancies	**1.31***	1.30*<br>(-2%)	1.30*<br>(-4%)	1.31*<br>(0%)	1.30*<br>(-2%)	1.30*<br>(-1%)	1.29*<br>(-7%)	1.30*<br>(-3%)	1.29*<br>(-9%)	1.28*<br>(-14%)	1.28*<br>(-15%)	1.30*<br>(-4%)	**1.25*<br>(-30%)**
Myeloid malignancies	**1.22***	1.20*<br>(-18%)	1.20*<br>(-16%)	1.22*<br>(0%)	1.20*<br>(-16%)	1.22*<br>(-1%)	1.20*<br>(-10%)	1.18*<br>(-26%)	1.21*<br>(-6%)	1.18*<br>(-32%)	1.18*<br>(-27%)	1.19*<br>(-19%)	**1.12*<br>(-66%)**
Breast	**0.95***	0.97*<br>(-59%)	0.95*<br>(-23%)	0.95*<br>(-1%)	0.97*<br>(-58%)	0.95*<br>(-4%)	0.95*<br>(-24%)	0.94*<br>(+21%)	0.96*<br>(-49%)	0.96*<br>(-37%)	0.93*<br>(+77%)	0.95*<br>(+5%)	**0.96*<br>(-40%)**
Uterus	**0.98**	0.97<br>(+204%)	0.97<br>(+108%)	0.98<br>(+25%)	0.98<br>(+38%)	0.97<br>(+173%)	1.01<br>(-50%)	1.01<br>(-81%)	0.95<br>(+665%)	0.97<br>(+108%)	0.80*<br>(+12812%)	0.95<br>(+467%)	**0.84*<br>(+7995%)**
Ovary	**0.97**	0.97<br>(+38%)	0.97<br>(+27%)	0.97<br>(+0%)	0.99<br>(-69%)	0.97<br>(+63%)	0.97<br>(-4%)	0.97<br>(+6%)	0.97<br>(+60%)	0.97<br>(+61%)	0.96<br>(+133%)	0.97<br>(+44%)	**0.96<br>(+182%)**
Cervix	**1.51***	1.47*<br>(-15%)	1.40*<br>(-33%)	1.51*<br>(-1%)	1.44*<br>(-22%)	1.52*<br>(+0%)	1.62*<br>(+32%)	1.45*<br>(-21%)	1.52*<br>(+1%)	1.45*<br>(-20%)	1.47*<br>(-15%)	1.47*<br>(-14%)	**1.39*<br>(-36%)**
Oropharynx	**1.50***	1.47*<br>(-8%)	1.45*<br>(-16%)	1.49*<br>(-2%)	1.48*<br>(-7%)	1.50*<br>(0%)	1.47*<br>(-8%)	1.35*<br>(-43%)	1.53*<br>(+10%)	1.40*<br>(-29%)	1.54*<br>(+12%)	1.45*<br>(-14%)	**1.40*<br>(-31%)**
Oesophagus, squamous	**1.19**	1.17<br>(-23%)	1.16<br>(-27%)	1.19<br>(-5%)	1.19<br>(-4%)	1.19<br>(-1%)	1.18<br>(-14%)	1.09<br>(-77%)	1.19<br>(0%)	1.10<br>(-72%)	1.34*<br>(+170%)	1.17<br>(-20%)	**1.20<br>(+3%)**
Oesophagus, adenocarcinoma	**1.30***	1.24<br>(-35%)	1.23<br>(-36%)	1.29*<br>(-6%)	1.28*<br>(-13%)	1.29*<br>(-5%)	1.31*<br>(+3%)	1.21<br>(-48%)	1.24<br>(-32%)	1.15<br>(-73%)	1.16<br>(-69%)	1.25*<br>(-27%)	**1.04<br>(-97%)**
Stomach	**1.37***	1.27*<br>(-41%)	1.28*<br>(-37%)	1.36*<br>(-5%)	1.33*<br>(-17%)	1.37*<br>(-3%)	1.35*<br>(-9%)	1.29*<br>(-35%)	1.34*<br>(-15%)	1.25*<br>(-48%)	1.33*<br>(-21%)	1.31*<br>(-27%)	**1.14<br>(-83%)**
Colorectum	**0.83***	0.83*<br>(-4%)	0.83*<br>(+5%)	0.83*<br>(+1%)	0.83*<br>(-1%)	0.83*<br>(0%)	0.83*<br>(-6%)	0.82*<br>(+18%)	0.84*<br>(-8%)	0.82*<br>(+10%)	0.82*<br>(+12%)	0.82*<br>(+12%)	**0.82*<br>(+13%)**
Pancreas	**1.22***	1.18*<br>(-26%)	1.19*<br>(-22%)	1.21*<br>(-3%)	1.20*<br>(-13%)	1.21*<br>(-2%)	1.20*<br>(-12%)	1.15*<br>(-47%)	1.21*<br>(-7%)	1.15*<br>(-52%)	1.18*<br>(-27%)	1.19*<br>(-19%)	**1.10<br>(-76%)**
Liver	**1.36***	1.29*<br>(-29%)	1.29*<br>(-31%)	1.35*<br>(-5%)	1.32*<br>(-19%)	1.36*<br>(-2%)	1.34*<br>(-7%)	1.30*<br>(-27%)	1.32*<br>(-17%)	1.26*<br>(-42%)	1.24*<br>(-51%)	1.30*<br>(-27%)	**1.11<br>(-88%)**
Biliary Tract	**1.21**	1.16<br>(-39%)	1.17<br>(-31%)	1.20<br>(-7%)	1.20<br>(-11%)	1.20<br>(-6%)	1.21<br>(+3%)	1.17<br>(-30%)	1.19<br>(-19%)	1.15<br>(-46%)	1.11<br>(-69%)	1.18<br>(-23%)	**1.05<br>(-93%)**
Malignant melanoma	**0.88***	0.94<br>(-72%)	0.93<br>(-69%)	0.88*<br>(-3%)	0.91<br>(-43%)	0.89*<br>(-9%)	0.88*<br>(+2%)	0.91<br>(-46%)	0.91<br>(-44%)	0.94<br>(-78%)	0.90*<br>(-23%)	0.92<br>(-56%)	**1.01<br>(-99%)**
Brain	**1.10**	1.10<br>(+14%)	1.09<br>(-14%)	1.10<br>(+7%)	1.10<br>(+7%)	1.09<br>(-7%)	1.09<br>(-18%)	1.10<br>(+3%)	1.10<br>(+7%)	1.10<br>(+12%)	1.09<br>(-15%)	1.10<br>(+10%)	**1.09<br>(-7%)**
Kidney	**1.30***	1.25*<br>(-25%)	1.26*<br>(-22%)	1.29*<br>(-3%)	1.26*<br>(-21%)	1.30*<br>(-1%)	1.26*<br>(-20%)	1.25*<br>(-24%)	1.25*<br>(-24%)	1.21*<br>(-46%)	1.19*<br>(-55%)	1.25*<br>(-24%)	**1.08<br>(-91%)**
Bladder	**1.15**	1.10<br>(-48%)	1.09<br>(-59%)	1.15<br>(-1%)	1.13<br>(-22%)	1.15*<br>(+1%)	1.14<br>(-14%)	1.05<br>(-88%)	1.14<br>(-16%)	1.04<br>(-93%)	1.12<br>(-36%)	1.10<br>(-51%)	**1.00<br>(-100%)**
Thyroid	**1.17**	1.16<br>(-11%)	1.19<br>(+21%)	1.17<br>(-4%)	1.16<br>(-11%)	1.17<br>(-2%)	1.15<br>(-27%)	1.20<br>(+26%)	1.15<br>(-24%)	1.17<br>(-2%)	1.14<br>(-34%)	1.17<br>(-9%)	**1.12<br>(-51%)**

Hazard ratio of cancer associated with rheumatoid arthritis: effect of adjustment
Percentages show reduction of chi-squared value attributable to rheumatoid arthritis compared to minimal adjustment. Asterisks (*) mark risks where p<0.05.

Figure S1. Never-smokers

Adjusted hazard ratio (HR) of cancer in individuals with rheumatoid arthritis (RA) compared to others in the Million Women Study, among never-smokers only. Risks were adjusted for year of9 recruitment; year of birth; 10 regions of residence; age; social deprivation; education; alcohol consumption; body mass index; strenuous exercise; age at menarche; age at first birth and parity; duration of oral contraceptive use; menopausal status and age; use of menopausal hormone therapy. Number of asterisks (*) indicates level of statistical significance (* <0.05; ** <0.005; *** <0.0005; **** <0.00005; *****<0.000005)

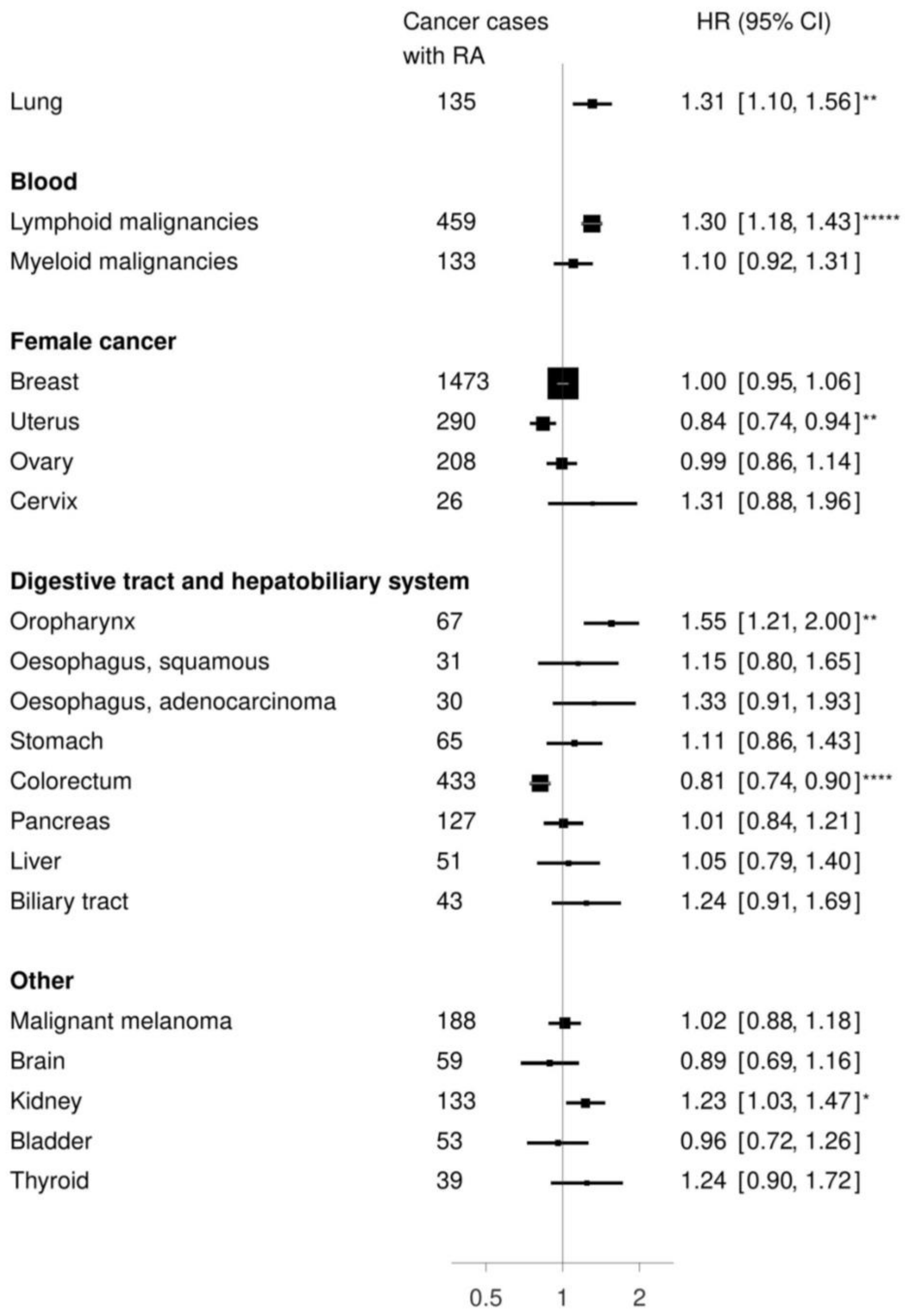

Figure S2. Excluding individuals who reported also having osteoarthritis

Sensitivity analysis: adjusted hazard ratio (HR) of cancer in individuals who reported having rheumatoid arthritis (RA) , excluding individuals who reported also having osteoarthritis, compared to individuals who did not report having rheumatoid arthritis in the Million Women Study. Risks were adjusted for year of recruitment; year of birth; 10 regions of residence; age; social deprivation; education; joint alcohol consumption and smoking status; body mass index; strenuous exercise; age at menarche; age at first birth and parity; duration of oral contraceptive use; menopausal status and age; use of menopausal hormone therapy. Number of asterisks (*) indicates level of statistical significance (* <0.05; ** <0.005; *** <0.0005; **** <0.00005; *****<0.000005)

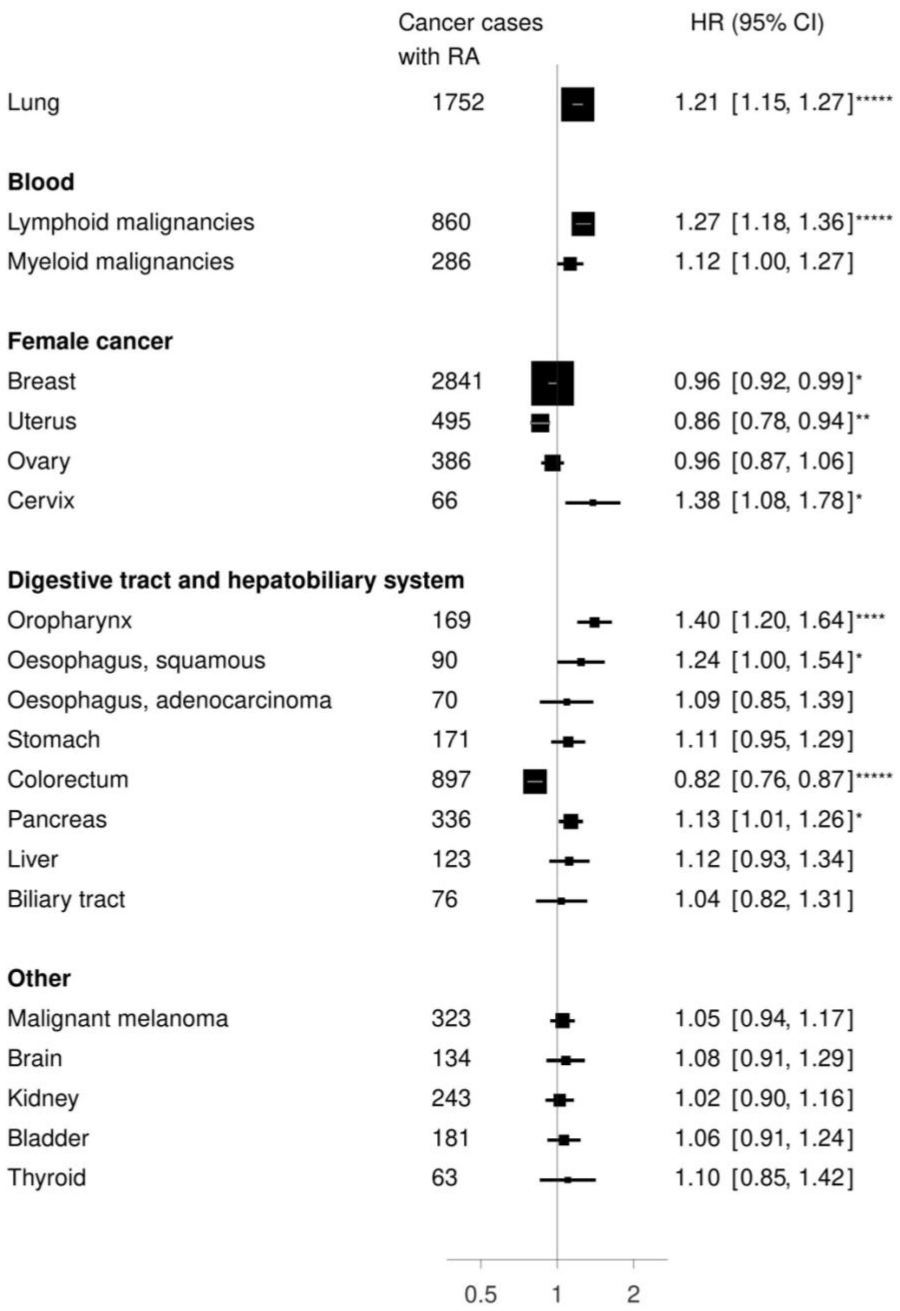

Figure S3. Excluding first five years of follow up

Adjusted hazard ratio (HR) of cancer in individuals with rheumatoid arthritis (RA) compared to others in the Million Women Study, excluding first five years of follow up. Risks were adjusted for year of recruitment; year of birth; 10 regions of residence; age; social deprivation; education; joint alcohol consumption and smoking status; body mass index; strenuous exercise; age at menarche; age at first birth and parity; duration of oral contraceptive use; menopausal status and age; use of menopausal hormone therapy. Number of asterisks (*) indicates level of statistical significance (* <0.05; ** <0.005; *** <0.0005; **** <0.00005; *****<0.000005)

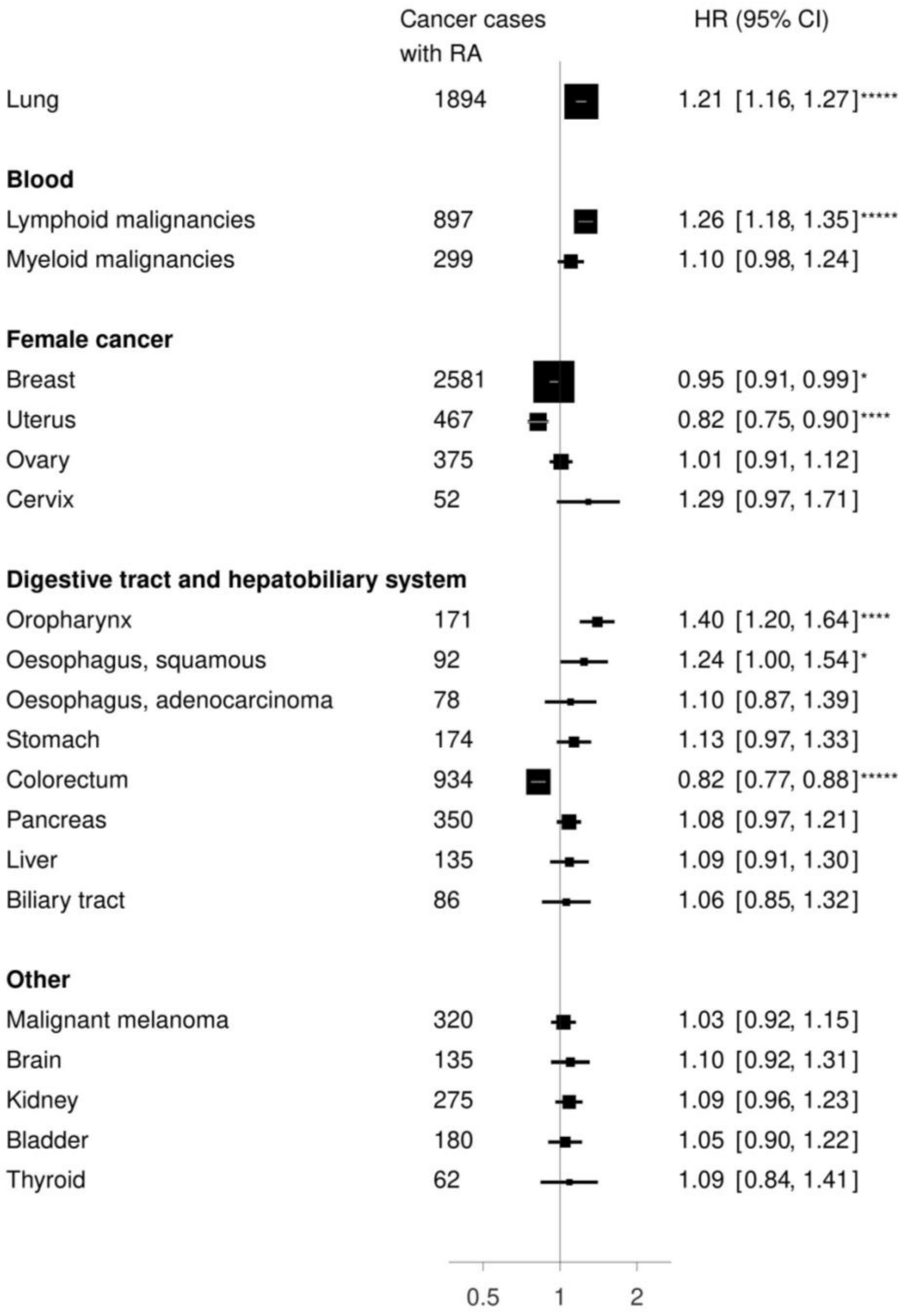

Figure S4. Excluding participants who had ever used menopausal hormone therapy

Adjusted hazard ratio (HR) of hormone and reproductive cancer in individuals with rheumatoid arthritis (RA) compared to others in the Million Women Study, excluding participants who had ever used menopausal hormone therapy. Risks were adjusted for year of recruitment; year of birth; 10 regions of residence; age; social deprivation; education; joint alcohol consumption and smoking status; body mass index; strenuous exercise; age at menarche; age at first birth and parity; duration of oral contraceptive use; menopausal status and age. Number of asterisks (*) indicates level of statistical significance (* <0.05; ** <0.005; *** <0.0005; **** <0.00005; *****<0.000005)

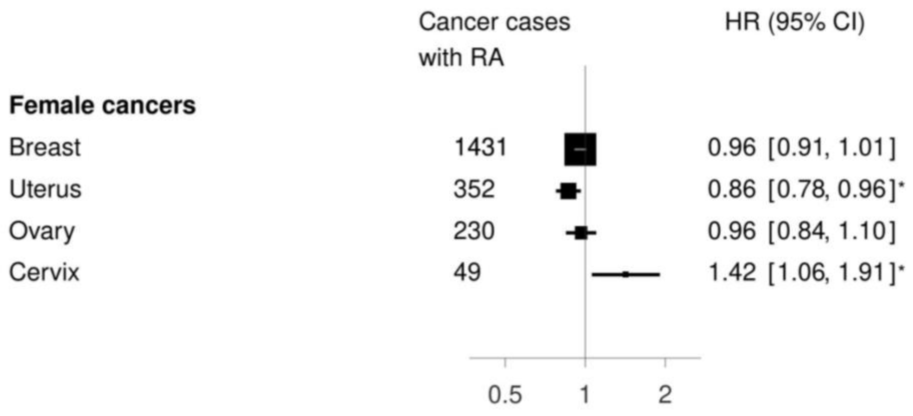

Figure S5. Excluding participants with missing information

Adjusted hazard ratio (HR) of cancer in individuals with rheumatoid arthritis (RA) compared to others in the Million Women Study, <u>excluding participants with missing information.</u> Risks were adjusted for year of recruitment; year of birth; 10 regions of residence; age; social deprivation; education; joint alcohol consumption and smoking status; body mass index; strenuous exercise; age at menarche; age at first birth and parity; duration of oral contraceptive use; menopausal status and age; use of menopausal hormone therapy. Number of asterisks (*) indicates level of statistical significance (* <0.05; ** <0.005; *** <0.0005; **** <0.00005; *****<0.000005)

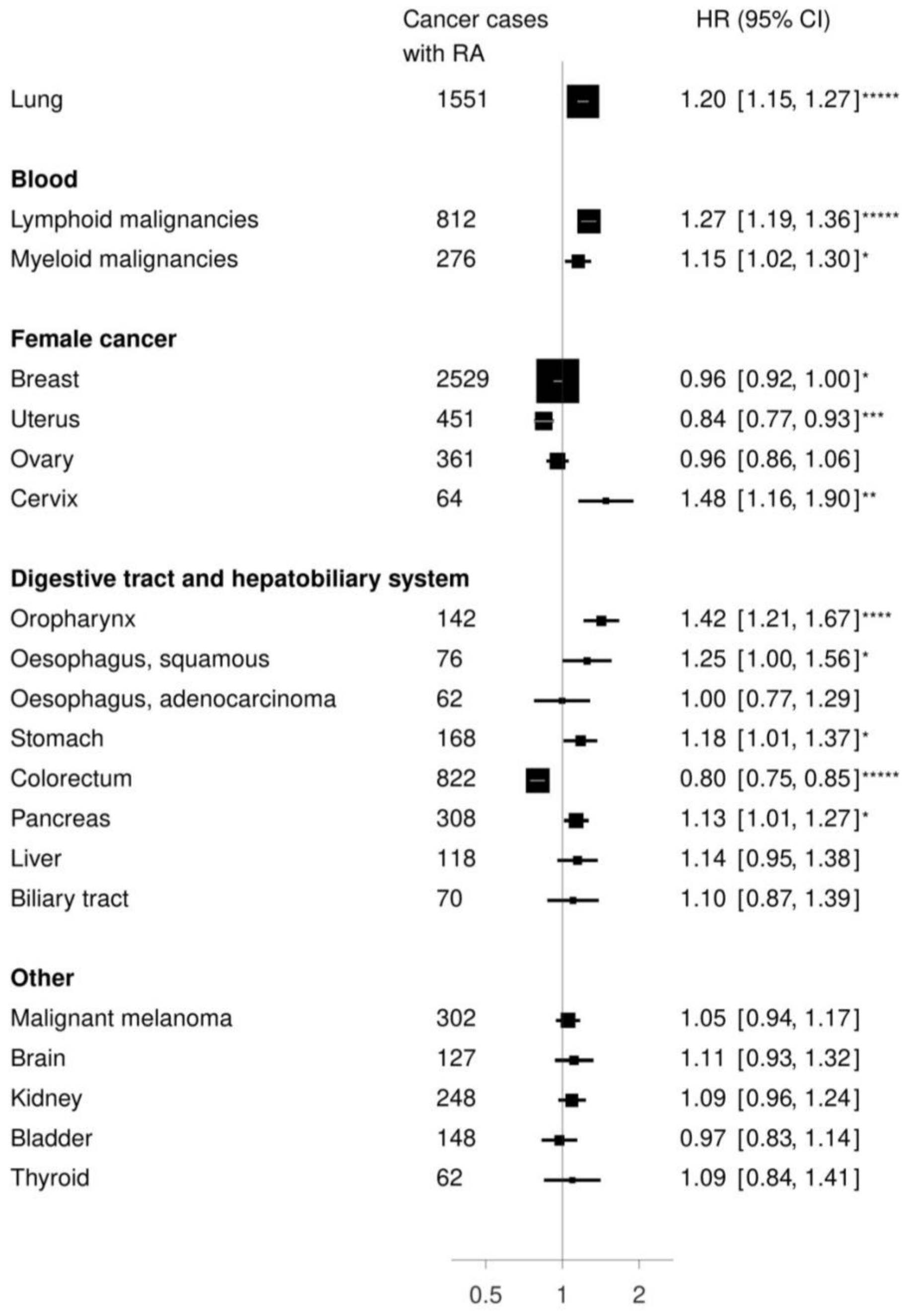

Figure S6. Use of alternative definition of self-reported rheumatoid arthritis which additionally requires a diagnosis of rheumatoid arthritis in hospital records

Sensitivity analysis: adjusted hazard ratio (HR) of cancer in individuals who reported having rheumatoid arthritis (RA), restricting to cases with a diagnosis of rheumatoid arthritis in hospital records, compared to individuals who did not report having rheumatoid arthritis in the Million Women Study. Risks were adjusted for year at recruitment; year of birth; 10 regions of residence; age; social deprivation; education; joint alcohol consumption and smoking status; body mass index; strenuous exercise; age at menarche; age at first birth and parity; duration of oral contraceptive use; menopausal status and age; use of menopausal hormone therapy. Number of asterisks (*) indicates level of statistical significance (* <0.05; ** <0.005; *** <0.0005; **** <0.00005; *****<0.000005)

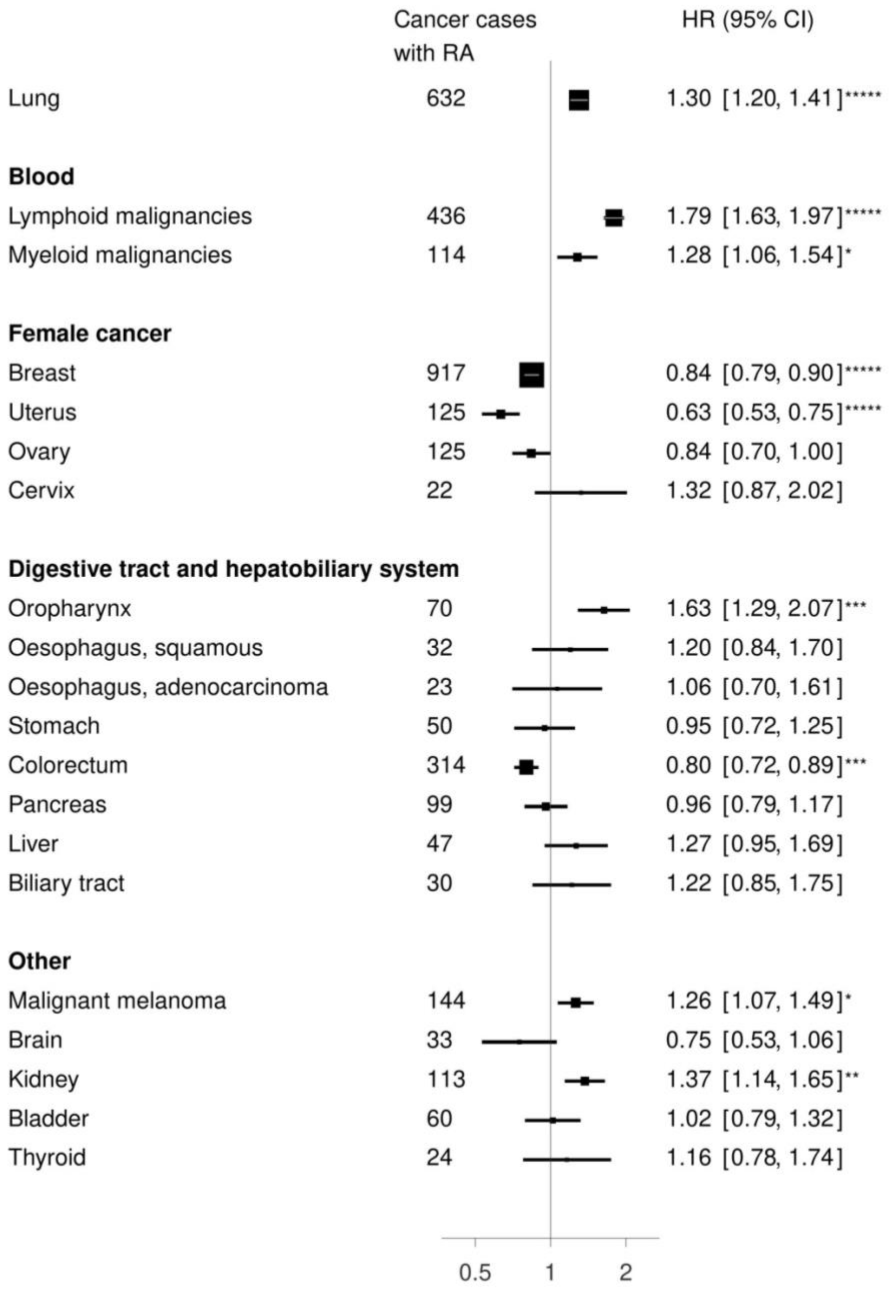

Supplement: dyae006_Supplementary_Data [file dyae006_supplementary_data.pdf]
